# Asian Americans & Racism: Individual and Structural Experiences (ARISE) pilot study: Feasibility of harmonizing data collection and blood collection for linguistically and culturally diverse Asian Americans for Alzheimer's Disease research

**DOI:** 10.1002/alz70858_104502

**Published:** 2025-12-25

**Authors:** Annie Ching, Oanh L. Meyer, Josh D Grill, Bora Nam, Marian Tzuang, Yirou Jiang, Daren Huang, Gabriel Fara‐On, Sarah Tomaszewski Farias, Boon Lead Tee, Quyen Q Tiet, Haeok Lee, Gilbert Gee, Marcelle Dougan, Thu T Nguyen, Bharat Thyagarajan, Michele Tobias, Van Ta Park, ARISE Pilot Study

**Affiliations:** ^1^ University of California San Francisco School of Nursing, San Francisco, CA, USA; ^2^ University of California, Davis School of Medicine, Sacramento, CA, USA; ^3^ The UC Irvine Institute for Memory Impairments and Neurological Disorders, Irvine, CA, USA; ^4^ Memory and Aging Center, UCSF Weill Institute for Neurosciences, University of California, San Francisco, San Francisco, CA, USA; ^5^ California School of Professional Psychology at Alliant International University, San Francisco, CA, USA; ^6^ Meyers College of Nursing, New York University, New York, NY, USA; ^7^ University of California at Los Angeles, Los Angeles, CA, USA; ^8^ San José State University, San Jose, CA, USA; ^9^ University of Maryland School of Public Health, College Park, MD, USA; ^10^ University of Minnesota, Minneapolis, MN, USA; ^11^ University of California, Davis, Davis, CA, USA; ^12^ Asian American Research Center on Health (ARCH), University of California San Francisco, San Francisco, CA, USA

## Abstract

**Background:**

Asian Americans (ASAs) are one of the fastest growing racial groups in the U.S. since 2000, with population projections surpassing 24 million by 2030. However, ASAs remain significantly underrepresented in research, including in Alzheimer's Disease (AD) studies. The ARISE study aims to assess the prevalence of multi‐level discrimination experiences among ASAs and its association on cognitive performance and AD biomarkers, while evaluating psychosocial protective factors and cardiometabolic risk factors as potential moderators. ARISE also aims to demonstrate feasibility of harmonizing data collection and blood collection for linguistically and culturally diverse ASA populations for AD research, which is described here.

**Method:**

ARISE, launched on May 15, 2024, has three sites with the goal of recruiting 500 older (≥65 years old) Chinese, Korean, and Vietnamese Americans in California. We developed a comprehensive study protocol and a centralized data management system. Each participant completes sociodemographic surveys (measuring social support, individual‐level discrimination, degree of acculturation), cognitive battery (measuring global cognition, memory, executive function), physical and mental health assessments (measuring cardiometabolic factors and depression) and a blood draw (measuring cardiometabolic and AD biomarkers) with data collection packets available in English, Chinese, Korean, and Vietnamese. Area‐level discrimination measures are derived from participants’ geographic data.

**Result:**

As of January 23, 2025, 252 (84 Chinese, 56 Korean, and 112 Vietnamese) participants have enrolled and completed the assessments, with 217 (86.1%) also having completed the blood draw (remaining participants are awaiting scheduling). The mean age of the participants is 74.37 (SD = 5.56) with 59% identifying as female. Nearly all are first‐generation (99%), most reported having limited English proficiency (85%), and 53% have attended some higher education. Recruitment is anticipated to conclude in June 2025, with subsequent processing and analysis of AD biomarkers to follow.

**Conclusion:**

The ARISE study will provide a better understanding of the frequency, severity, and implications of multi‐level discrimination on cognitive performance and as a social determinant of AD pathophysiology. ARISE's study design and recruitment demonstrate the feasibility of engaging more ASAs into AD research, and establishes a robust framework for protocols and procedures in multilingual cognitive research for future studies.

